# Characterization of perfusion decellularized whole animal body, isolated organs, and multi‐organ systems for tissue engineering applications

**DOI:** 10.14814/phy2.14817

**Published:** 2021-06-29

**Authors:** Doris A. Taylor, Stefan M. Kren, Katrina Rhett, Matthew J. Robertson, Jacquelynn Morrissey, Osman E. Rodriguez, Hassan Virk, Lourdes Chacon‐Alberty, Ernesto Curty da Costa, Fernanda C. P. Mesquita, Luiz C. Sampaio, Camila Hochman‐Mendez

**Affiliations:** ^1^ Regenmedix Consulting LLC Houston TX 7030 USA; ^2^ Lillehei Heart Institute Univeristy of Minnesota USA; ^3^ Department of Physical Therapy and Occupational Therapy Idaho State University USA; ^4^ Advanced Technologies Cores Baylor College of Medicine Houston USA; ^5^ Regenerative Medicine Research Texas Heart Institute USA; ^6^ Division of Infectious Diseases University of Texas Houston USA; ^7^ Center for Advanced Cardiopulmonary Therapies and Transplantation University of Texas Health Science Center Houston USA

**Keywords:** decellularization, extracellular matrix, whole organ engineering, whole rat

## Abstract

To expand the application of perfusion decellularization beyond isolated single organs, we used the native vasculature of adult and neonatal rats to systemically decellularize the organs of a whole animal in situ. Acellular scaffolds were generated from kidney, liver, lower limb, heart‐lung system, and a whole animal body, demonstrating that perfusion decellularization technology is applicable to any perfusable tissue, independent of age. Biochemical and histological analyses demonstrated that organs and organ systems (heart‐lung pair and lower limb) were successfully decellularized, retaining their extracellular matrix (ECM) structure and organ‐specific composition, as evidenced by differences in organ‐specific scaffold stiffness. Altogether, we demonstrated that organs, organ systems and whole animal bodies can be perfusion decellularized while retaining ECM components and biomechanics.

## Graphical abstract

Representative images from systemic perfusion decellularization of an adult female whole rat body. Histological staining of excised organs and organs systems shows the absence of cells yet maintenance of tissue‐specific characteristics.

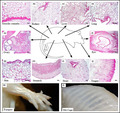

## 1 INTRODUCTION

Bioengineering whole organ replacements remains a promising strategy for addressing the worldwide shortage of donor organs. The first critical step for building complex solid organs is establishing a relevant biological scaffold on which cells can be incorporated. Naturally derived extracellular matrix (ECM) scaffolds can be generated by removing cells from a tissue or organ using decellularization (He & Callanan, 2013). Historically, decellularization techniques involved immersing a tissue or organ into a detergent solution (Badylak, 2007; Gilbert et al., 2006) to remove all of the cellular components. Successful decellularization using various physical, chemical, or enzymatic protocols have been described in the literature for organs and tissues ranging from heart valves (Elkins et al., 2001) to hollow (Atala et al., 2006; Totonelli et al., 2012) and solid organs (Uygun et al., 2010; Ott et al., 2008) to nerves (Szynkaruk et al., 2013). However, conventional decellularization techniques are efficient only for very thin tissues that are just a few cell layers thick (Koenig et al., 2019). We have previously used perfusion decellularization to create a whole heart ECM scaffold (Ott et al., 2008). Since its first report in 2008, perfusion decellularization has been extensively studied in ex vivo perfusion of isolated organs (Mendibil et al., 2020). This technique generates a cell‐free scaffold called decellularized ECM (dECM), which is used broadly in cell biology and tissue engineering research, including studies of ECM composition and biophysical properties, and scaffold recellularization.

Effective tissue decellularization is dictated by factors such as tissue density and organization and geometric and biologic properties in addition to the need to preserve the complex composition and three‐dimensional ultrastructure of the dECM (Crapo et al., 2011). ECM is an intricate network composed of an array of multidomain macromolecules organized in a cell/tissue‐specific manner. ECM components link together to form a structurally stable composite, contributing to the mechanical properties of tissues. It is now evident that ECM is a highly dynamic entity (Bissell & Aggeler, 1987) and its physiological relevance extends beyond simple structural integrity, determining and controlling cell adhesion, proliferation, migration, polarity, differentiation, and apoptosis (Bonnans et al., 2014; Hynes, 2009; Lu et al., 2011). There is evidence that the decellularization process can alter the composition of the ECM and influence residual cellular components (Fernandez‐Perez & Ahearne, 2019; Fischer et al., 2017), diminishing the biological scaffold's constructive tissue remodeling capacities. However, perfusion decellularization when performed with immersion bypasses the issue of solution penetration to the inner part of the scaffold without compromising the native vasculature complexity.

Here, we demonstrate the perfusion decellularization of a whole rat body as a proof of concept that this technology can decellularize any perfusable solid organ in situ. Currently, the ability to decellularize scaffolds from different organs in situ remains limited (Gerli et al., 2018; Kajbafzadeh et al., 2015). Using both harvested (traditional) and non‐harvested (in situ) perfusion, decellularized organs showed the preservation of the vascular conduits, organ capsules, ECM components, and structural architecture, and presented low levels of DNA, while preserving the glycosaminoglycan, chemical and mechanical components of the ECM. We demonstrate organ‐specific differences in mechanical properties and molecular composition in decellularized tissues.

By validating the efficiency of our decellularization protocol at the chemical, ultrastructural, and tissue level, we demonstrate that this method is feasible for generating quality scaffolds of single organs (liver, kidney, heart, and lung), combined organ systems (heart‐lung pair), lower limb, and whole animals. The adaptability of our simple, modular and scalable protocol allows small adjustments in the decellularization reagents and duration to be made to effectively decellularize organs of varying age. Moreover, our approach to decellularizing a whole animal body in situ results in blocks of preserved organ systems which can be used for investigations of tissue engineered heart‐lung systems and other organ pairings.

## 2 METHODS

### 2.1 Animals and anesthesia

All experiments were performed in accordance with NIH guidelines for the care and use of laboratory animals and the US Animal Welfare Act, and were approved by the Institutional Animal Care and Use Committee at the University of Minnesota (#0806A37501) and at Texas Heart Institute (#HSC‐AWC‐12–122). Adult Sprague Dawley rats (230–305 g) (Harlan Labs) were anesthetized with ketamine/xylazine (100/10 mg/kg body weight) (Phoenix Pharmaceutical) via systemic heparinization injected into femoral or intragastric vein. For whole neonatal animal studies, 2–5‐day‐old Sprague Dawley rats (Harlan Labs) were euthanized with CO_2_ without heparinization.

### 2.2 Whole rat decellularization

An appropriately sized catheter was introduced into the carotid and femoral arteries and advanced as far as possible toward the aortic arch in euthanized adult and neonatal rats. After cannulation, a small hole was made in the inferior vena cava to allow fluid and cell debris to exit followed by perfusion of the whole animal with 1% sodium dodecyl sulfate (SDS) in deionized water at 80 mm Hg. Perfusion rates and pressures were driven by gravity and hydrostatic pressure gradients. Perfusion was temporarily stopped when the visceral organs were visually translucent to remove heart, lung, liver, kidney and other internal organs. The vascular attachments of the internal organs were ligated prior to organ harvesting to avoid leakages that could affect the perfusion of the remaining tissues. Perfusion was then continued until the skeletal muscle mass was translucent. Times varied from 7 to 9 days depending on the animal size, cannulation efficiency, and vessel preservation. After obtaining translucent organs, tissues were washed with deionized water followed by phosphate saline buffer (PBS).

### 2.3 Perfusion decellularization of excised organs and organ systems

To decellularize different types of organs used in this study we adapted our previously reported perfusion decellularization protocols (Hochman‐Mendez et al., 2020; Ott et al., 2008). The renal artery of kidney, the portal vein of liver, the descending aorta of heart and lung, and the iliac artery of the thigh (gracilis cranialis and the gluteus superficialis) were cannulated and perfused with 1% SDS. The organs were washed with deionized water, 1% Triton‐X100 (Sigma), and PBS containing 100 U/ml penicillin‐G and 100 U/ml streptomycin (Life Technologies). Next, the scaffolds, with the exception of skeletal muscle, were perfused with recirculating DNase (Roche, 100 U/mL of buffer) for 4 h at 25°C followed by washing with recirculating antibiotic‐containing PBS.

### 2.4 Dye perfusion

Decellularized individual organs and organ systems were perfused with either methyl blue or blue dextran at 10 mg/ml in saline. After dye injection, the flow through the vascular conduits was recorded using a Canon HV20 (Canon USA, Inc.) with a diopter macro lens (Raynox). The clips were edited in Final Cut Express (Apple, Inc.).

### 2.5 Biochemical Assays

DNA content was quantified using Quan‐iT^™^ PicoGreen^™^ dsDNA assay kit (Invitrogen) and GAG was quantified using Blyscan^™^ Glycosaminoglycan assay kit (Blyscan, #B1000), according to the manufacturers’ instructions, as previously described (Taylor et al., 2018). Briefly, for DNA analysis, cadaveric and decellularized tissues were digested with 1 N NaOH in a heating block for 3 h at 65°C. Samples were neutralized with 10X Tris‐EDTA buffer, and the pH was adjusted to 7.0. Samples were plated in duplicate against a calf thymus standard prepared according to kit instructions and were read using a Tecan Infinite M200 Microplate Reader (Tecan Trading AG) set to 480 nm excitation and 530 nm emission. Percentage of residual DNA in decellularized organ was obtained by comparing DNA values of decellularized organs and systems to that in their respective native cadaveric organ. For GAG content, cadaveric and decellularized tissues were digested using NaOH hydrolysis. We hydrolyzed 20–300 mg of wet tissue in 2 ml 1 N NaOH, and then neutralized with TRIS and 1 N HCI. Percentage of residual sulfated GAG in decellularized organ was obtained by comparing values of all sulfated GAG concentrations of decellularized tissue to that in their respective native cadaveric organ.

### 2.6 Histology

Cadaveric and decellularized samples were formalin‐fixed (10% buffered formalin), paraffin‐embedded, and cut into 5‐μm sections. Sections were deparaffinized with xylene and then rehydrated using an ethanol dilution series with subsequent hematoxylin and eosin (H&E) and Masson's Trichrome staining, as previously described (Ott et al., 2008). Images were obtained using a Nikon Eclipse TE200 inverted microscope (Nikon).

### 2.7 Immunostaining

Paraffin‐embedded tissue sections were deparaffinized using xylene and rehydrated with graded alcohol. To detect ECM proteins, antigens were retrieved at 95°C for 20 min using Tris‐EDTA Buffer (10 mM Tris Base, 1 mM EDTA, 0.05% Tween 20, pH 9.0) then blocked with blocking buffer (5% Normal Donkey Serum, Sigma‐Aldrich #566460) at room temperature for 30 min. Primary antibodies were incubated overnight and include: rabbit anti‐collagen type I (Abcam, #ab34710, 1:200 dilution) and rabbit anti‐elastin (Abcam, #ab21610, 1:100 dilution). After washing in PBS with 0.05% Tween 20 (3 times), the slides were incubated in secondary antibody (Alexa Fluor^®^ 594 AffiniPure Donkey Anti‐Rabbit IgG [H + L], #711‐515–152, Jackson ImmunoResearch, Inc.) at 1:200 in blocking buffer for 1 h at room temperature. The slides were then mounted with Vectashield mounting medium (Vector Laboratories) for fluorescence with DAPI.

### 2.8 Scanning electron microscopy

Scanning electron microscopy (SEM) was performed, as previously described (Ott et al., 2008). Briefly, cadaveric and decellularized tissues were fixed with 2.5% (v/v) glutaraldehyde in a buffered solution of 0.1 M sodium cacodylate buffer (pH 7.3) and post‐fixed with 1% osmium tetroxide buffered with 0.1 M sodium cacodylate (pH 7.3). The specimens were dehydrated, dried, and sputter‐coated with 10 nm AuPd (60%/40% alloy). Images were obtained using a JEOL 5310 SEM (JEOL USA, Inc.).

### 2.9 Mechanical testing

Decellularized heart, kidney, lung, and liver tissues were sectioned into rostro‐caudal and lateral strips (longitudinal and circumferential direction), and clamp‐mounted to a tensile mechanical testing machine (Instron) and submerged in PBS. Thickness, length between the two clamps, and width of the sample were measured by caliper. Samples were preconditioned by six cycles of loading and unloading with the upper strain limit of 0.25 and stretched until failure. Strain rate was 0.01 s^−1^ throughout the test. Stress was calculated by normalizing force values to the original cross‐sectional area. Strain was defined as the ratio of clamp displacement to the sample's original length. Ultimate tensile strength (UTS) was defined as maximal stress before failure. Young's modulus was calculated as the slope of the linear region in the stress‐strain curve, and membrane tension was calculated as the product of UTS and thickness.

### 2.10 Proteomic analysis of decellularized tissue

The left ventricle of three decellularized rat hearts, the cortex and medulla of three decellularized rat kidneys, and the left lateral lobe of three decellularized rat livers were placed into 1 ml of deglycosylation solution containing 0.05 U of Heparinase II from *Flavobacterium* *heparinum*, 0.05 U of Chondroitinase ABC from *Proteus vulgaris*, and 0.05 M sodium acetate in 1 ml of PBS with Ca^2+^/Mg^2+^ with 10 µL of protease inhibitor cocktail. Tissue was minced using micro‐scissors and placed onto a rotating platform for 2 h at room temperature. After centrifugation, supernatant was removed, and the deglycosylated tissues were rinsed twice using 1 ml of PBS with Ca^2+^/Mg^2+^. The samples were analyzed at the Proteomics Core Laboratory at the University of Texas MD Anderson Cancer Center. Briefly, samples were digested in 5% trypsin overnight at 37°C, and the peptides were separated using liquid chromatography‐tandem mass spectrometry (LC‐MS). Probable protein matches were analyzed using the MASCOT search engine with 2 allowed missed trypsin cleavages, variable modifications, and a SwissProt database search (Rodentia taxonomy with 26138 sequences). In MASCOT, significance threshold was set at *p* < 0.05. The resulting protein chain data were used to identify all ECM components. For each decellularized organ, the presence of a protein was only reported when identified in at least two out of three replicates.

### 2.11 Statistical analysis

Data are shown as mean ±standard deviation. Comparison between cadaveric and scaffold groups for nucleic acid content was performed using Student *t*‐test. All other data were analyzed using two‐way ANOVA with Bonferroni post hoc test and *p* < 0.05 was considered significant. Statistical analyses were performed using GraphPad Prism^®^, version 7.0 (GraphPad Software, Inc.) and SPSS.

## 3 RESULTS

### 3.1 Systemic adult whole animal decellularization

**FIGURE 1:**
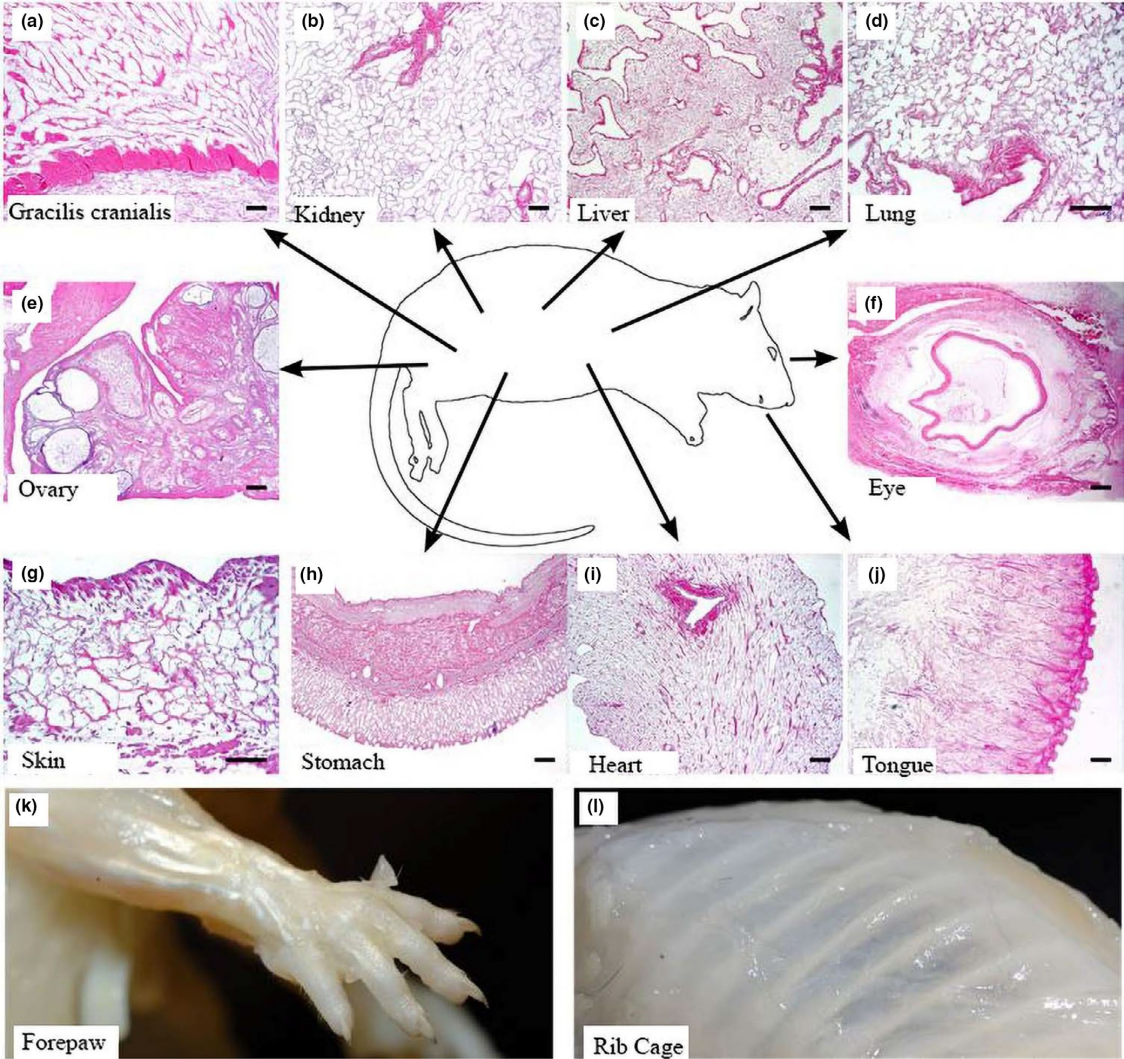
Decellularization of whole adult female rat. (a–j) H&E representative staining of gracilis cranialis (a), kidney (b), liver (c), lung (d), ovary (e), eye (f), skin (g), stomach (h), heart (i) and tongue (j). Representative pictures of decellularized adult rat forelimb and paw with 5 digits (k), and decellularized adult rat thoracic vertebrae with ribcage (l). Scale bar represents 50 µm

To examine the application of perfusion decellularization beyond isolated single organs, we used the native vasculature of an adult rat to systemically decellularize the organs of a whole animal in situ. A whole adult rat was decellularized (Figure 1) and histological examination demonstrated a good level of decellularization—that is, no cells detected, and maintenance of the tissue‐specific structures in the gracilis cranialis (Figure 1a), kidney (Figure 1b), liver (Figure 1c), lung (Figure 1d), ovaries (Figure 1e), eye (Figure 1f), stomach wall (Figure 1h), heart (Figure 1i), and tongue (Figure 1j). Developing follicles were observed in ovaries (Figure 1e), and longitudinal, circular, and oblique muscle layers with gastric pit‐like structures on the luminal surface were observed in stomach (Figure 1h). The tongue surface retained taste bud‐like structures (Figure 1j). However, some distant tissues like skin still presented some remaining cellular debris (Figure 1g and Figure S1). Finally, complete decellularization of skeletal muscles was visualized by the muscles in the forepaw (Figure 1k) and the intercostal muscles of the rib cage (Figure 1l).

**FIGURE 2:**
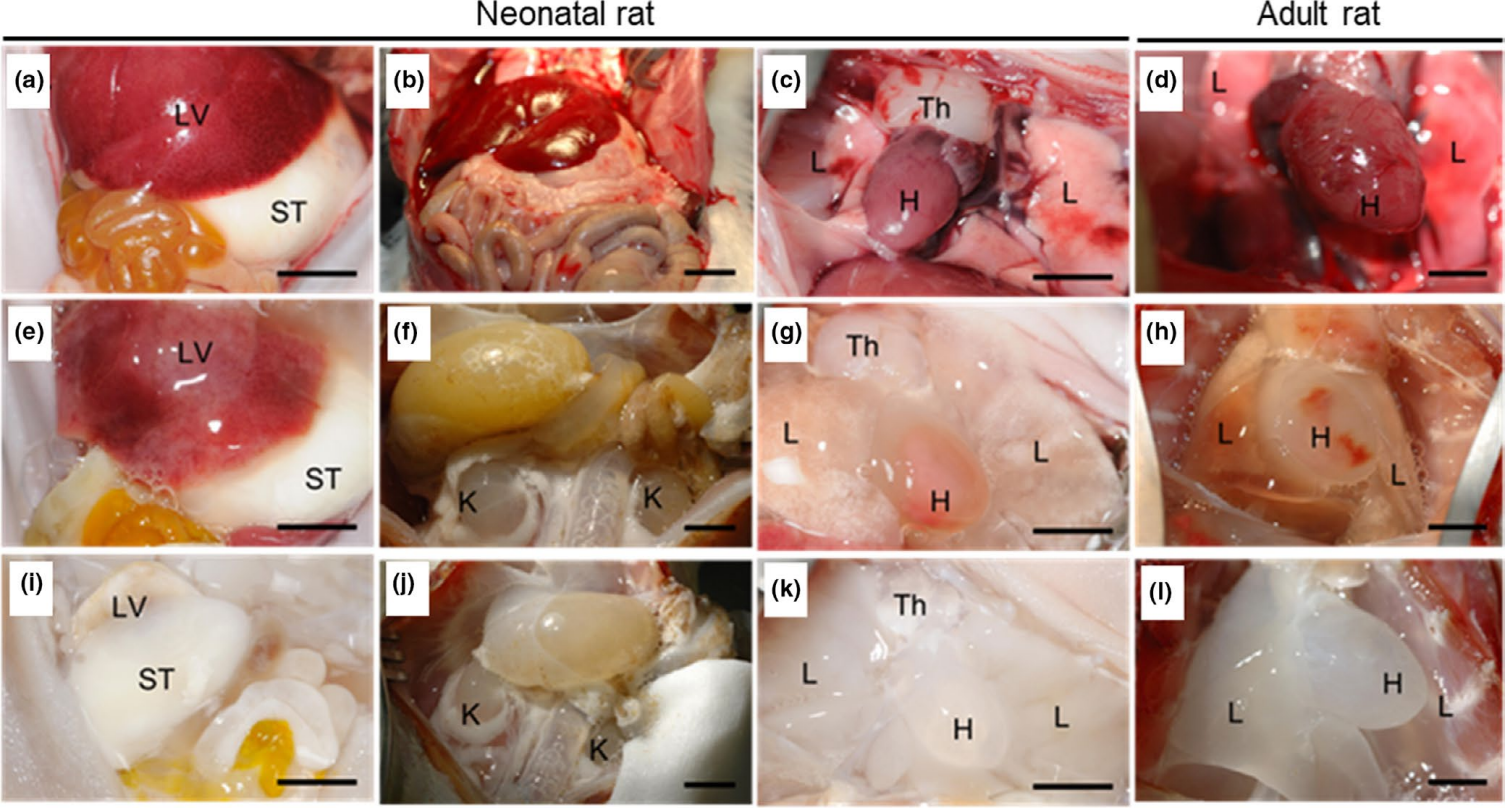
Whole animal decellularization of neonatal and adult rats. (a–l) Representative images depict the decellularization process of cadaveric tissue in the abdominal compartment (a, b) and thoracic compartment (c) of a neonatal rat and the thoracic compartment (d) of an adult rat, through intermediate stage of cellular removal (e–h), to completion of decellularization (i–l). H, heart; K, kidney; L, lung; LV, liver; ST, stomach; Th, thymus. Scale bar = 5 mm

The stages of the decellularization process both in neonatal (2–5‐days; Figure 2a–c,e–g,i–k) and adult (Figure 2d,h,l) rats reveals that organs can be successfully decellularized by whole‐body systemic perfusion. Using the systemic vasculature, we demonstrated that abdominal (liver, stomach and kidney, Figure 2a,b,e,f,i,j) and thoracic compartments (thymus, heart and lung, Figure 2c,g,k) in both neonatal and adult rats were visually decellularized at the end of the procedure.

### 3.2 Characterization of decellularized organs and organ systems

**FIGURE 3:**
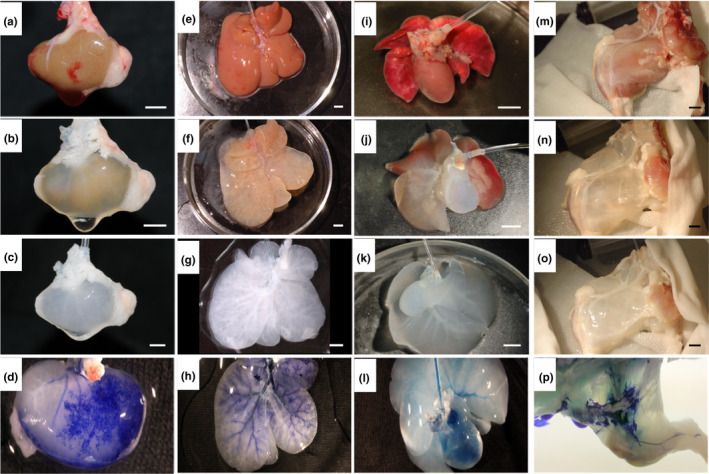
Perfusion decellularization of organs and organs system. (a–o) Representative images of decellularization process of kidney (a, b, c), liver (e, f, g), heart‐lung pair (i, j, k) and lower limb (m, n, o); Representative images of mid‐decellularization (b, f, j, n); Representative images of decellularized organs (c, g, p) and organs system (k). Representative image of dye‐perfusion (d, h, l, p). Scale bar: 5 mm

After demonstrating the ability of using systemic vasculature to guide the decellularization solutions in a whole animal body, we performed ex vivo perfusion of individual organs (kidney and liver; Figure 3a–d,e–h, respectively) and organ systems (heart‐lung pair and the whole lower limb; Figure 3i–l,m–p, respectively). A timeline shows cadaveric (Figure 3a,e,i,m), mid (Figure 3b,f,j,n), and late (Figure 3c,g,k,o) stages of the decellularization process. At the end of the decellularization, all organs (both isolated or within organ systems) showed a characteristic translucent appearance. These data were supported by the quantification of residual DNA of these decellularized organs (Table 1). Perfusion of dye through the scaffolds outlined the remaining vascular network and demonstrated preservation of organ capsule (Figure 3d,h,l,p and Video S1). It is important to highlight that the cardiothoracic system (heart‐lung pair) was dissected and harvested while maintaining the connective circulatory system (pulmonary veins and arteries); this allowed preservation of the connective circulatory system after decellularization as demonstrated by presence of dye in both lungs after retrograde aortic dye perfusion (Figure 3l and Video S2).

**TABLE 1:** Quantification of residual DNA and glycosaminoglycans (GAGs)

Decellularized organ	*n*	% DNA content versus cadaveric	GAG content (µg/mg wet weight)
Heart	6	2.474 ± 1.562	0.055 ± 0.026
Lung	13	0.988 ± 0.627	0.089 ± 0.043
Kidney	9	1.525 ± 1.680	2.247 ± 0.014
Liver	4	2.141 ± 1.223	0.554 ± 0.120

Data represents mean ± standard deviation.

**FIGURE 4:**
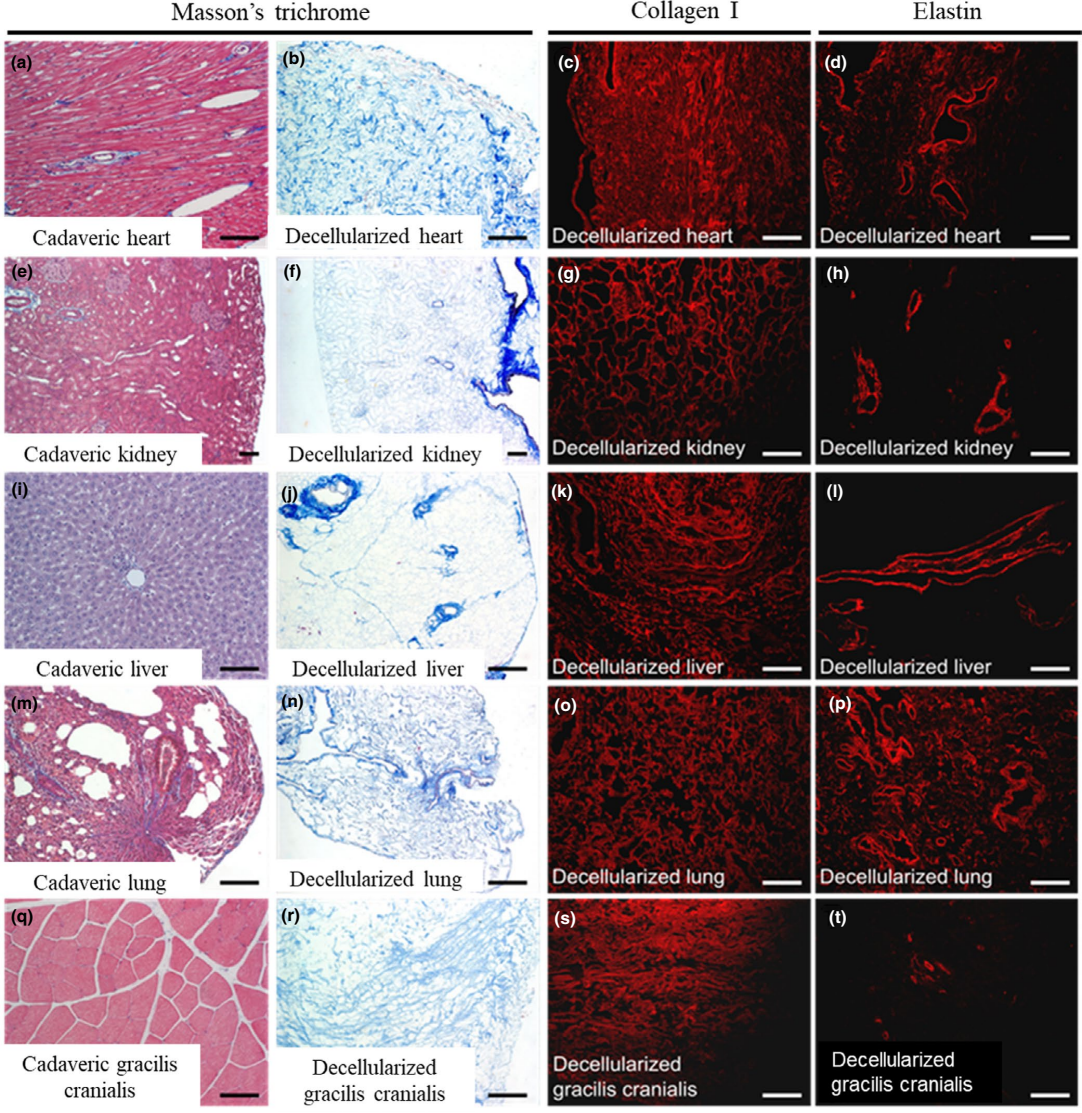
Immunohistochemical characterization of decellularized organs. Heart (a–d); kidney (e–h); liver (i–l); lung (m–p); gracilis cranialis (q–t). The two left columns represent a bright field image of a Masson's trichrome staining of cadaveric and decellularized tissues. The third and fourth columns show immunofluorescence staining for collagen I and elastin in decellularized tissues. Scale bars: 5 mm (Masson's trichrome) and 100 µm (Collagen I and Elastin)

To confirm the efficiency of the decellularization process at the tissue level, we performed histological evaluations with Masson's trichrome staining in the isolated organs and organ systems (Figure 4). As observed in the whole animal assays (Figures 1 and 2), the cellular component and nuclei present in the cadaveric sections (Figure 4a,e,i,m,q) were removed from the scaffold after decellularization (Figure 4b,f,j,n,r), while the collagen composition was preserved (Figure 4c,g,k,o,s). Elastin staining (Figure 4d,h,l,p,t) confirmed the maintenance of vessels in decellularized organs.

**FIGURE 5:**
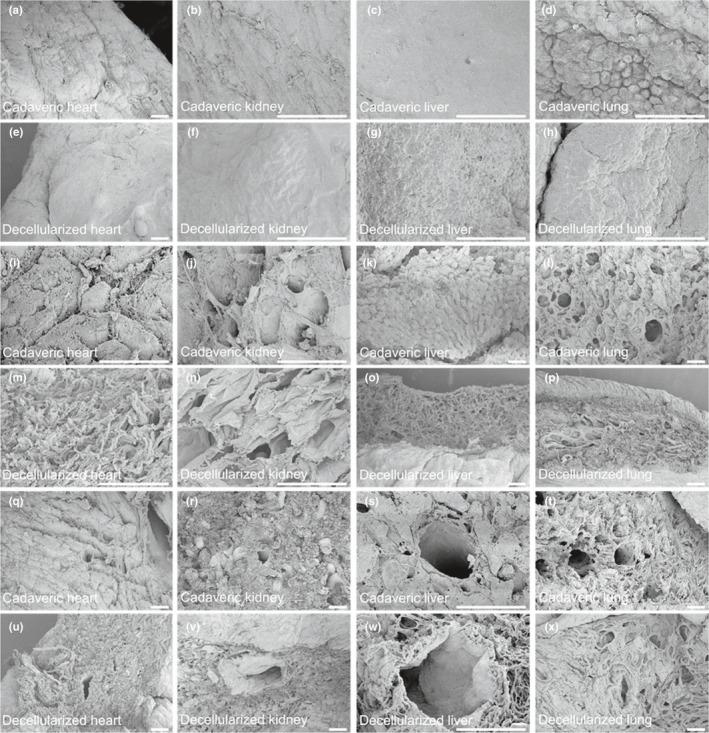
Scanning electron micrographs of decellularized organs. Representative images of cadaveric (a–d) and decellularized (e–h) capsules;and cadaveric (i–l) and decellularized (m–p) parenchyma of heart, kidney, liver, and lung. Representative image of the vasculature within the parenchyma of cadaveric (q–t) and decellularized (u–x) organs. Scale bar: 50 µm

Ultrastructure was investigated via comparison of representative SEM images of decellularized hearts, lungs, kidneys, and livers with that of cadaveric organs (Figure 5). ECM fiber orientation (Figure 5m,n,o,p) was preserved in decellularized organs in addition to preservation of the epicardial wall in heart, the capsules in kidney and liver, and the external surface of lung (Figure 5e,f,g,h, respectively). Decellularized vessel lumens and basement membranes were visible (Figure 4u,v,w,x), corroborating the elastin staining observed in Figure 3. The matrix of all organs was highly preserved, and specific structures such as kidney glomeruli were easily identified even after decellularization (Figure 3B and Figure S2).

To chemically characterize the dECM from different organs, we performed LC‐MS‐based proteomics on rat decellularized hearts, kidney and liver and performed MASCOT search for data analyses. We identified structural (collagen, elastin, fibrillin, and fibronectin), basement membrane (collagen, laminin, and perlecan), cell‐ECM interaction (microfibril‐associated glycoprotein 4, periostin, and TGFβIp), and fiber‐regulating (lumican) proteins in all decellularized organs (Table 2). As compared to decellularized heart and liver, kidney showed more basement membrane proteins (~30% additional types) comprised mainly by the presence of different laminin isoforms. We detected the presence of ECM‐associated peroxidases in decellularized kidney only.

**TABLE 2:** Residual extracellular matrix proteins in decellularized heart, kidney, and liver

	Decellularized heart	Decellularized kidney	Decellularized liver
Structural proteins	Collagen 1	Collagen 1	Collagen 1
	Collagen 3	–	Collagen 3
	Collagen 5	–	Collagen 5
	Collagen 6	Collagen 6	Collagen 6
	–	Collagen 12	Collagen 12
	–	–	Collagen 14
	Elastin	Elastin	Elastin
	Fibrillin‐1	Fibrillin‐1	Fibrillin‐1
	Fibronectin	Fibronectin	Fibronectin
Basement membrane proteins	Collagen 4	Collagen 4	Collagen 4
	Collagen 15	–	–
	–	Collagen 18	–
	–	Laminin‐111	–
	Laminin‐221	–	–
	–	Laminin‐411	–
	–	Laminin‐421	–
	Laminin‐511	Laminin‐511	Laminin‐511
	Laminin‐521	Laminin‐521	Laminin‐521
	Nidogen‐2	Nidogen‐2	–
	Perlecan	Perlecan	Perlecan
	–	vWF A domain‐containing protein	–
Cell‐ECM interaction proteins	Fibulin‐3	–	Fibulin‐3
	Microfibril‐associated glycoprotein 4	Microfibril‐associated glycoprotein 4	Microfibril‐associated glycoprotein 4
	Periostin	Periostin	Periostin
	TGFbIp	TGFbIp	TGFbIp
Fiber‐regulating proteins	Lumican	Lumican	Lumican
	–	Biglycan	–
ECM‐associated peroxidases	–	Peroxidasin homolog	–

*n* = 3 for each group.

Abbreviations: ECM, extracellular matrix; TGFβIp, transforming growth factor‐β‐induced protein ig‐h3; vWF, von Willebrand factor.

We quantified the mechanical properties of decellularized heart, lung, kidney, and liver ECM scaffolds using a biaxial tensile test in two directions (circumferential and longitudinal). The UTS and the stiffness (Young's modulus) are shown in Table 3. For each organ, directionality did not impact the mechanical properties of the tissue, with the exception of isolated liver. The UTS and Young's modulus for the heart and liver were higher—in the range of 1000–2000 kPa—than that (below 300 kPa) for the kidney and lung. We observed a statistically significant difference (*p* = 0.0006) between circumferential and longitudinal measurements in liver. Overall, considering the variance of the data, our study is underpowered to draw conclusions.

**TABLE 3:** Mechanical properties of decellularized organs

Decellularized organ	*n*	Ultimate tensile strength (kPa)	Young's Modulus (kPa)
Heart			
Circumferential	4	1814.4 ± 565.2	2647.4 ± 957.9
Longitudinal	4	1055.6 ± 463.3	2172.8 ± 652.2
Lung			
Circumferential	7	209.7 ± 46.4	288.5 ± 88.7
Longitudinal	7	189.1 ± 55.8	233.6 ± 98.0
Kidney			
Circumferential	4	83.0 ± 50.4	167.3 ± 45.0
Longitudinal	4	91.9 ± 34.3	178.9 ± 50.2
Liver			
Circumferential	9	1652.1 ± 394.6	2728.7 ± 569.4
Longitudinal	9	1548.6 ± 307.3	4242.2 ± 891.6

Data represents mean ± standard deviation.

The heart and lung were decellularized together as a complete system.

## 4 DISCUSSION

Here, we demonstrated that perfusion decellularization is a simple, effective, and universal technique that can generate organ scaffolds from any vascularized tissue. Using either ex vivo or in situ perfusion, decellularized organs showed the preservation of the vascular conduits, organ capsules, ECM components, and structural architecture, and presented low levels of DNA, while preserving the glycosaminoglycan, chemical and mechanical components of the ECM. By validating the efficiency of our decellularization protocol at the chemical, ultrastructural, and tissue level, we have demonstrated that this method is feasible for generating quality scaffolds of single organs (liver, kidney, heart, and lung), combined organ systems (heart‐lung pair and lower limb), and, to a lesser extent, whole animals. Using a similar perfusion‐based technique, other researchers have achieved whole animal decellularization to generate numerous organ scaffolds from a single animal (Kajbafzadeh et al., 2015). However, to the best of our knowledge, this is the first description of whole animal body decellularization in neonatal and adult rats.

We report that perfusion decellularization of whole rats is feasible but not optimal. We adapted our whole heart decellularization protocol consistent with it taking more time. To ensure perfusion throughout the furthest reaches of the whole carcass, we stopped the decellularization process when major internal organs were visually translucent and restarted the perfusion after harvesting these organs. However, distant tissues like the skin retained some observable cellular debris. The longer decellularization time had a deleterious effect on the delicate vasculature in the mesentery and viscera. Vessel integrity broke down in the rat leg, resulting in leaking during perfusion with dye. Variation in the preparations made the whole animal body method impossible to precisely standardize.

Perfusion decellularization of single organs and organ pairs using our adapted protocol resulted in biologically active whole organ scaffolds with preserved ECM components and biomechanics. Using liquid chromatography–tandem mass spectrometry (LC–MS), we demonstrated that the decellularized scaffolds from distinct organs preserved their organ‐specific ECM composition, including native collagen composition. Although decellularized heart, kidney, and liver scaffolds contained collagen 1 and collagen 6, we found differences in the content of collagens 3, 5, 12, and 14. Collagens 3 and 5 were found in heart and liver but not in kidney, whereas collagen 12 was found in liver and kidney but not in heart. Collagen 14 was found exclusively in the liver. Although we did not examine collagen cross‐linking, we postulate that increased stiffness (Young's modulus and UTS) of the heart and liver compared with the lung and kidney may be due, in part, to different heterotypic fiber formations and collagen content, as observed by the presence of collagen 3 and 5 in heart and liver and absence in the kidney, which may be cross‐linked with collagen 1.

We found that decellularized kidneys had more variety of basement membrane proteins than livers and hearts such as collagen 18, laminin‐111, laminin‐411, laminin‐421, and von Willebrand factor type A domain‐containing protein. Basement membrane is composed mainly of collagen 4, laminin, nidogen and proteoglycans (such as perlecan, collagen 15 and collagen 18). The presence of several isoforms of laminin in kidney is due the glomerulus basement membrane (Pollak et al., 2014), maintained even after the decellularization process. The presence of these basement membrane proteins indicates that our decellularization protocol maintains the organ‐specific architecture of the ECM, and can facilitate cell recognition and attachment during the recellularization process.

When comparing the results of the proteomic analysis of our decellularized scaffolds with previous reports on ECM composition for cadaveric organs, we found that most ECM proteins identified in our heart scaffolds were present in cadaveric hearts, aside from fibulin‐3, TGFblp, and perlecan (Doll et al., 2017). For the kidney and liver scaffolds, all proteins reported here were consistent with previous data on the ECM composition of respective cadaveric organs (Doll et al., 2017; Hobeika et al., 2017). However, the qualitative nature of the proteomics assay that we employed does not permit determination of the relative abundance of these proteins maintained in our decellularized scaffolds.

In sum, we demonstrate the ability to generate scaffolds derived from organ systems, instead of only isolated organs, that preserve organ‐specific biochemical and biophysical characteristics. The simplicity, versatility, and universal applicability of perfusion decellularization in vascularized tissues allows for the streamlined generation of biologically pertinent whole organ scaffolds.

## CONFLICTS OF INTEREST

Dr. Doris A. Taylor holds a financial interest in Miromatrix Medical, Inc. and is entitled to sales royalty through the University of Minnesota for products related to the research described in this paper. This relationship has been reviewed and managed by the University of Minnesota and Texas Heart Institute in accordance with its conflict of interest policies. All other authors have nothing to disclose with regard to commercial support.

## AUTHOR CONTRIBUTIONS

Conception and design of the study (DAT, SMK, KR, MJR); data collection (SMK, KR, MJR); analyses and interpretation (DAT, SMK, KR, MJR, CHM, LCS, FCPM, ECC, JM, OER, HV, LCA); writing/critical revision of the manuscript for important intellectual content (CHM, LCS, FCPM, ECC, JM, OER, HV, LCA).

## Supporting information



Figures S1–S2

Video S1

Video S2
