# Supplementary material for: Characterization of perfusion decellularized whole animal body, isolated organs, and multi‐organ systems for tissue engineering applications
Source: Physiol Rep. 2021 Jun 29;9(12):e14817. doi: 10.14814/phy2.14817 (PMC8239446; doi:10.14814/phy2.14817)
Supplement: Supplementary file 1 — Figures S1–S2 [file PHY2-9-e14817-s002.pptx]

## Slide 1
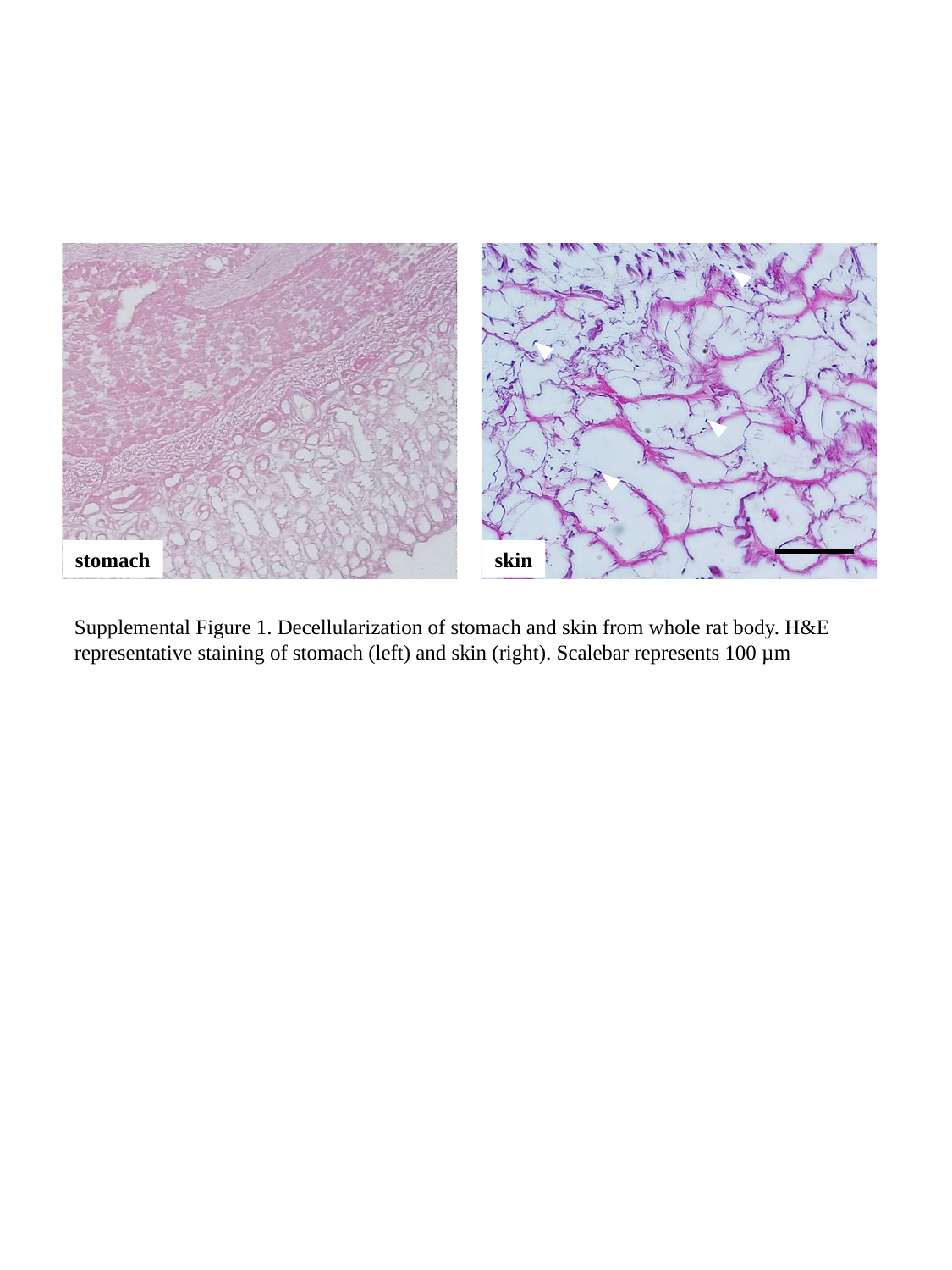

stomach
skin
Supplemental Figure 1. Decellularization of stomach and skin from whole rat body. H&E representative staining of stomach (left) and skin (right). Scalebar represents 100 µm

## Slide 2
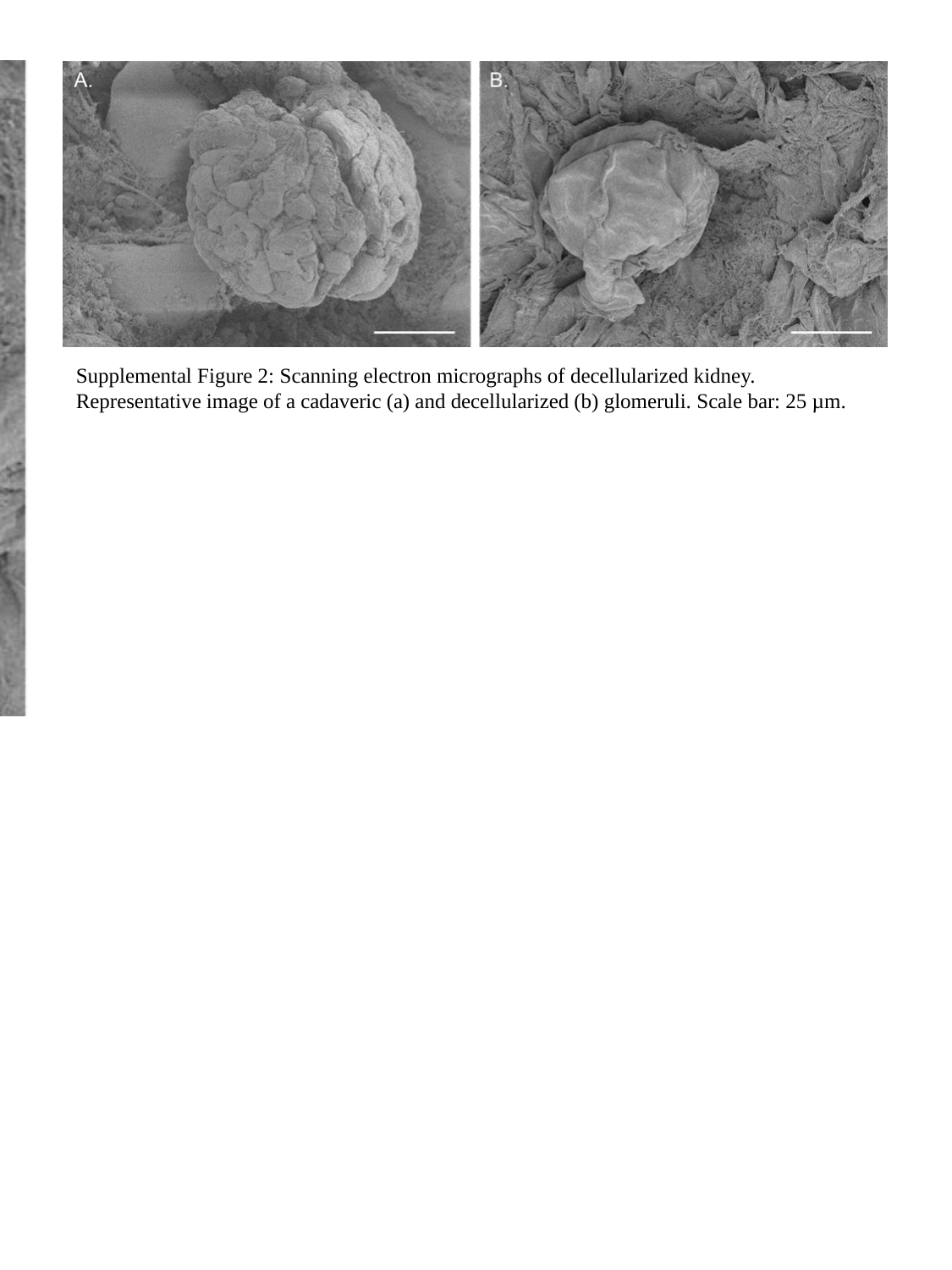

Supplemental Figure 2: Scanning electron micrographs of decellularized kidney. Representative image of a cadaveric (a) and decellularized (b) glomeruli. Scale bar: 25 µm.
